# Coproduction and Usability of a Smartphone App for Falls Reporting in Parkinson Disease

**DOI:** 10.1093/ptj/pzad076

**Published:** 2023-06-27

**Authors:** Jill Wales, Jason Moore, Jenni Naisby, Natasha Ratcliffe, Gill Barry, Annee Amjad, Alan Godfrey, Gerry Standerline, Elaine Webster, Rosie Morris

**Affiliations:** Department of Sport, Exercise and Rehabilitation, Northumbria University, Newcastle-upon-Tyne, UK; Department of Computer and Information Sciences, Northumbria University, Newcastle-upon-Tyne, UK; Department of Sport, Exercise and Rehabilitation, Northumbria University, Newcastle-upon-Tyne, UK; Parkinson’s UK, London, UK; Department of Sport, Exercise and Rehabilitation, Northumbria University, Newcastle-upon-Tyne, UK; Parkinson’s UK, London, UK; Department of Computer and Information Sciences, Northumbria University, Newcastle-upon-Tyne, UK; Person with Parkinson disease, Co-Researcher, UK; Person with Parkinson disease, Co-Researcher, UK; Department of Sport, Exercise and Rehabilitation, Northumbria University, Newcastle-upon-Tyne, UK

**Keywords:** Accidental Falls, App, Co-Production, Digital Measurement Tool, Parkinson Disease, Usability

## Abstract

**Objective:**

The purpose of this study was to coproduce a smart-phone application for digital falls reporting in people with Parkinson disease (PD) and to determine usability using an explanatory mixed-methods approach.

**Methods:**

This study was undertaken in 3 phases. Phase 1 was the development phase, in which people with PD were recruited as co-researchers to the project. The researchers, alongside a project advisory group, coproduced the app over 6 months. Phase 2 was the implementation phase, in which 15 people with PD were invited to test the usability of the app. Phase 3 was the evaluation phase, in which usability was assessed using the systems usability scale by 2 focus groups with 10 people with PD from phase 2.

**Results:**

A prototype was successfully developed by researchers and the project advisory group. The usability of the app was determined as good (75.8%) by people with PD when rating using the systems usability scale. Two focus groups (n = 5 per group) identified themes of 1) usability, 2) enhancing and understanding management of falls, and 3) recommendations and future developments.

**Conclusions:**

A successful prototype of the iFall app was developed and deemed easy to use by people with PD. The iFall app has potential use as a self-management tool for people with PD alongside integration into clinical care and research studies.

**Impact:**

This is the first digital outcome tool to offer reporting of falls and near-miss fall events. The app may benefit people with PD by supporting self-management, aiding clinical decisions in practice, and providing an accurate and reliable outcome measure for future research.

**Lay Summary:**

A smartphone application designed in collaboration with people who have PD to record their falls was acceptable and easy to use by people with PD.

## Introduction

Parkinson disease (PD) is a common neurodegenerative disorder that presents with both motor and nonmotor impairments. Falls are common in people with PD, who are 2 to 4 times more likely to fall when compared with age-matched older adults and those with other neurological conditions.^1^^,^^2^ Falls in people with PD are complex and multifactorial and lead to reduced quality of life, increased caregiver burden, reduced independence, and shorter life expectancy.^3–5^ Treatments for falls in this population are currently limited, and there has been recent drive from people with PD and their caregivers, family members, and friends to improve treatments for falls.^6^

In the research setting, paper-based falls diaries provide the gold standard method for recording falls incidence in people with PD^7^. Critically, the rate of falls often provides the primary outcome measure for large randomized controlled trials.^8^^,^^9^ Although the current gold standard, this type of reporting requires a significant resource to operate efficiently and can lead to high amounts of missing data and attrition.^7^ Furthermore, due to the retrospective nature of falls reporting, falls frequency is often underestimated due to inaccurate recall. In PD, underestimation of falls frequency may be exacerbated by the high frequency of falls and incidence of cognitive impairment, which is a common nonmotor symptom.^10^

In the clinical setting, understanding circumstances around fall events facilitates more effective rehabilitation and future fall prevention. People with PD are a heterogeneous population with a variety of factors relating to falls risk (eg, freezing of gait, balance impairment, postural hypotension, anxiety, and polypharmacy, to name only a few)^5^ and therefore effective interventions need to be tailored to each individual person with PD.^11^ Improving accuracy of falls reporting by including information regarding the fall event (eg, time of day, environment, and activity at time of fall) provides rich information for health care professionals to tailor individual falls management. Furthermore, longitudinal information about how fall patterns evolve over time in individuals would benefit falls management in the clinic.

Accurate falls reporting will benefit people with PD and health care professionals both in research and clinical settings. One way in which to overcome current challenges and ease the burden of falls reporting, is to develop and utilise a digital measurement tool for falls reporting, that is, an application (app) for smartphones and tablets. Providing an easy-to-use falls app would enable people with PD to promptly record a fall event, or near miss fall, which could be recorded immediately or soon after the event. Information recorded on the app has the potential to be shared remotely with health care professionals and be implemented into clinical practice. Furthermore, a falls reporting app has excellent potential to further inform clinical trials as a digital endpoint.

Development of a digital measurement tool lends itself to coproduction, in which stakeholders share the power of research, including research design, interpretation, and dissemination.^12^^,^^13^ The process of coproduction improves both the quality and relevance of research and has become a recent focus of the research community.^12^ For technology such as apps, stakeholder involvement in the process ensures the technology is suitable to the target population, with early understanding of user experience providing information which enhances the later development of a technological intervention.^14^ The opinion of people with lived experience of PD in the development of an app is vital to create the optimum tool that individuals with PD will find beneficial in their day-to-day lives. In addition, digital measures that are meaningful for people with PD are vital within clinical trials. Therefore, when creating digital measurement tools, a collaborative working group of researchers, people with PD, and their caregivers is pertinent.

The 3 aims of this project were to coproduce an app (iFall) for reporting fall events and near misses alongside a development group that included people with PD and caregivers, determine usability of the app by testing over 6 months using the System Usability Scale (SUS), and gain feedback on the iFall app from users with PD via semistructured interview focus groups.

## Methods

To achieve our aims, we used a 3-phase approach: phase 1 (development), in which coproduction was used to develop a prototype of the app; phase 2 (implementation), in which the acceptability and usability of the app were tested; and phase 3 (evaluation), in which feedback on the app was obtained to determine its use and development goals for subsequent versions. An explanatory sequential mixed-methods design was carried out using qualitative research to build and develop an understanding of quantitative results.^15^Supplementary Figure 1 shows a study overview.

### Phase 1: Development

The first step in the coproduction project was to recruit co–principal investigators (Co-PIs) who were people with PD; this was done by advertising for Co-PI roles via Parkinson’s UK. After Co-PIs (E.W. and G.S.) were recruited, the project team (R.M., E.W., and G.S) was formed. The project team then held discussions around the first prototype of the app. The app developer (J.M.) created an initial prototype based on feedback from the project team.

Once the initial prototype was developed, a project advisory group (PAG) was recruited via Parkinson’s UK. The 5 members of the PAG who were recruited were 2 people with PD and 3 family members/caregivers of people affected by PD. Over 6 months, 4 workshops were conducted with the project team, app developer, and PAG. Within the PAG workshops, the concept of the app was explained alongside an outline of what was to be achieved within the workshops. Members of the PAG were then asked to download the app prototype and asked to navigate the app and provide feedback. Feedback was integrated into a new version of the prototype app and later downloaded by the same PAG participants. This process continued until the project team and PAG were happy with the app prototype to be implemented in phase 2. Three iterations of the app were developed alongside the PAG prior to phase 2.

### Phase 2: Implementation

This implementation phase was undertaken to determine acceptability and usability of the app in people with PD over 6 months. The study was granted ethical approval from Northumbria University Ethics committee (Ethics ID 24868 and 35604).

#### Recruitment

Study participants were recruited from Parkinson’s UK. Recruitment criteria were as follows: a confirmed diagnosis of idiopathic PD by a movement disorder specialist, daily access to a smartphone, and English speaking and literate. People were excluded if they had significant cognitive impairment so that they could not understand instructions for using the app.

#### Beta-Testing

The app was developed to be compatible with Android smartphones. Android smartphones were issued to those participants without a suitable smartphone to allow for digital inclusion. Once participants had consented to the study, they were asked to download the iFall app to their device with the support of step-by-step guides. Participants were asked to use the app in their daily life and record a fall or near miss fall event if they occurred. During the development phase, definitions of a fall (“A fall is defined as an event which results in a person coming to rest inadvertently on the ground or floor or other lower level”) and a near miss (“A near miss is defined as a time when you thought you were going to fall but did not”) were developed alongside the PAG; these definitions were provided on the report screen on the app via the question mark icon. Participants used the iFall app for 6 months and were contacted once per month by the researcher to provide support if required. During the testing phase, all participants were given “dummy” falls to log on the app to ensure all participants experienced using the app.

### Phase 3: Evaluation

#### Testing Measurements

Following the 6-month implementation phase, participants were provided with the SUS. The questionnaire was modified for use in the iFall project (Tab. 1). The SUS is a tool that has been widely used to assess the usability of technologies.^16^

**Table 1:** System Usability Scale for Quantitative Feedback*^a^*

**Question**	**Usability**
1	I think I would like to use the iFall App frequently.
2	I found the iFall app unnecessarily complex.
3	I thought the iFall app was easy to use.
4	I think that I would need the support of a technical person to be able to use the iFall app.
5	I found the various functions in the iFall app were well integrated.
6	I thought there was too much inconsistency in the iFall app.
7	I would imagine that most people would learn to use the iFall app very quickly.
8	I found the iFall app to be very awkward to use.
9	I felt very confident using the iFall app.
10	I needed to learn a lot of things before I could get going with the iFall app.

^a^ The System Usability Scale was scored from 1 (strongly disagree) to 5 (strongly agree).

#### Focus Groups

Following the implementation phase, participants were invited to take part in focus groups to provide feedback on the participants’ use of the technology and experience of using the iFall app. Focus groups took place online via video calling platform (Microsoft Teams; Microsoft, Redmond, WA, USA), and consent was gained to record all calls. The focus groups were facilitated by both researchers and people with PD (R.M., E.W., G.S., and J.N.). Focus group schedules were developed by researchers and people with PD (R.M., E.W., and G.S.) to determine usability of the app, user experience, and clinical implications. See the Supplementary Appendix for the interview schedule. Each focus group discussion lasted approximately 75 minutes. Recordings were downloaded immediately following the meeting and were then transcribed verbatim by an independent transcriber.

#### Data Analysis

Data from the SUS were collated and summarised to provide quantitative feedback on the use of the app. The mean and SD of the total SUS score was calculated using SPSS v26 (IBM SPSS, Chicago, IL, USA). Transcripts from the focus groups were uploaded to NVivo 12 Pro (QSR International, Burlington, MA, USA) for the analysis. Framework analysis was used to analyse the focus group data.^17^ Framework analysis is a thematic analysis methodology that uses interrelated steps to assist the management of qualitative data and analysis.^18^ Two independent researchers (R.M. and J.N.) familiarized themselves with the data and coded the transcripts independently to develop a thematic framework. Subsequently, the researchers met to compare and discuss codes and refine the thematic framework and develop charts of the data into a Framework Matrix. Following this, data were interpreted, and themes were developed and refined. These meetings ensured both researchers perspectives were considered and captured within the data analysis. The final themes were then discussed with the coresearchers and further refined.

### Role of the Funding Source

The funder played no role in the design, conduct, or reporting of this study.

## Results

### Development Phase

#### Initial Prototype and PAG Meetings

**Figure 1:**
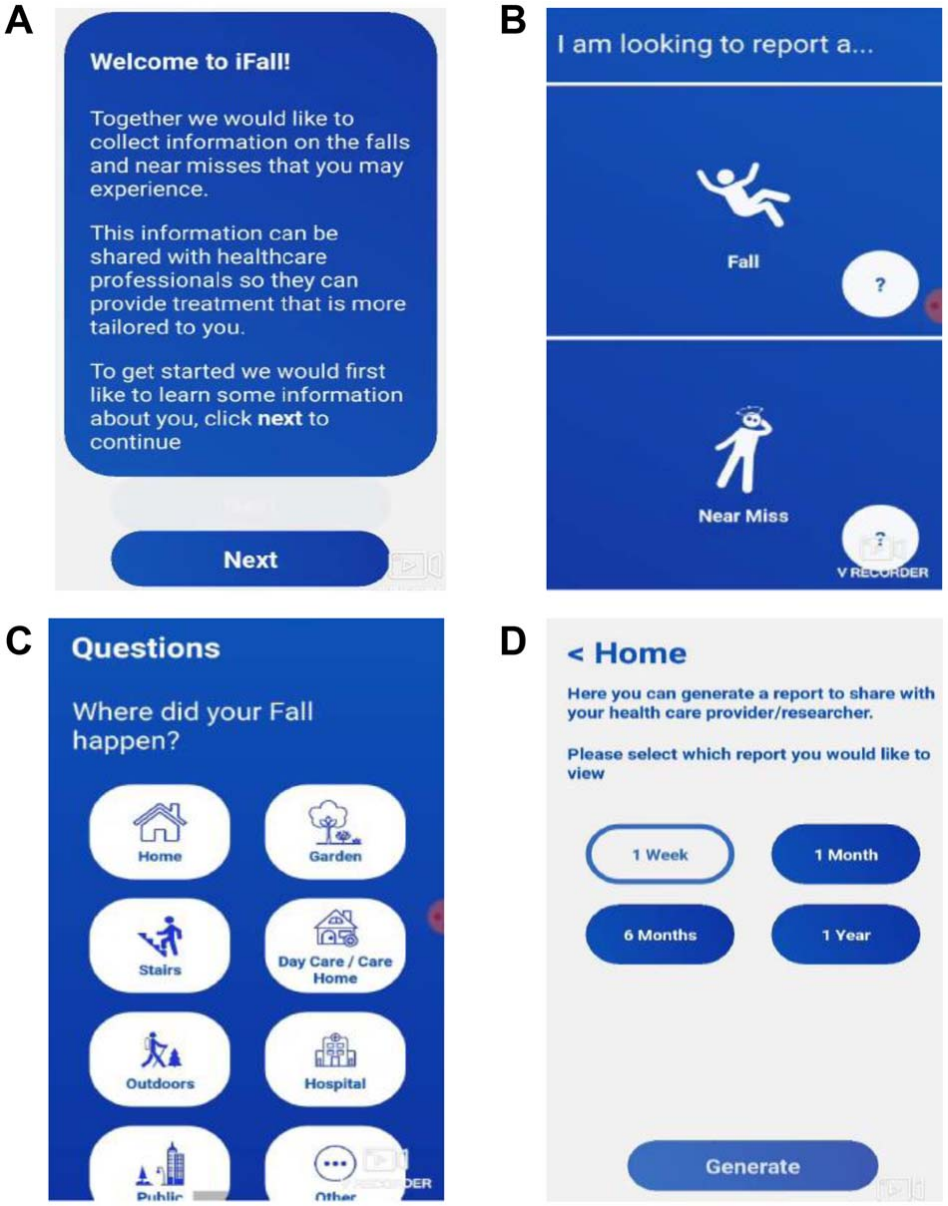
Screenshots of the iFall prototype. (A) Welcome screen. (B) Report screen. (C) Fall details. (D) Creation of a falls report.

Following initial meetings with the project team, an initial draft prototype was developed to present to the PAG. In the initial prototype, the design was minimal, information was mainly recorded via textboxes and there were limited options to select for falls location, cause of fall and injury reporting. Several meetings were held with the PAG to develop the first prototype that was rolled out within the beta-testing phase. Members of the PAG were introduced to the initial prototype and used the previous iterations of the app to provide feedback. Following use and feedback, over 6 months the design of the app was changed. For example, larger buttons were implemented, audio and tactile feedback was added, increased options were added for all screens, a calendar and clock function was implemented, and an emoji screen was also provided to detail feelings following a fall. Screenshots of the iFall prototype agreed for beta-testing by the PAG are shown in Figure 1.

#### Implementation and Evaluation Phases

**Figure 2:**
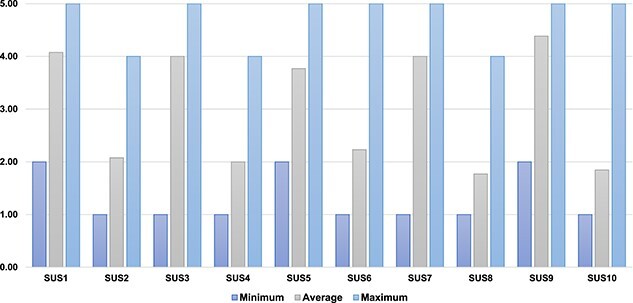
Participant responses to the System Usability Scale (SUS) questionnaire. Results are presented for the average as well as minimum and maximum responses to each question.

In total, 13 participants (8 men, 5 women; with a mean age of 69.6 [SD = 9.0] years and with a mean disease duration of 8.69 [SD = 6.21] years at the time of initial app download) downloaded the app to assess usability; Table 2 shows participant characteristics. A total of 13 participants completed the SUS questionnaire. The mean score on the SUS was 75.8% (SD = 19.3%), classified as good^16^; the average score and minimum and maximum scores for each question on the SUS can be found in Figure 2. Of the 13 participants who completed the SUS, 9 participants scored the SUS above 66.5 points, which is the average score for apps.^16^ Further results of specific questions of the SUS have been integrated into qualitative themes below to further support these findings.

**Table 2:** Study Participant Characteristics*^a^*

**Characteristic**	**Value in Study**	
	**1 (n = 13)**	**2 (n = 10)**
**Age, y, mean (SD)**	69.6 (9.0)	68.3 (8.7)
**Gender (reported as male/female sex)**	8/5	7/3
**Disease duration, y**	8.69 (6.21)	8.10 (5.70)
**Smartphone experience, no. (%)**	13 (100)	10 (100)
**Faller, yes/no**	9/4	7/3

^a^ Study 1 = quantitative analysis of using the smartphone app; study 2 = focus groups for qualitative feedback.

During the implementation phase, 84 true events were logged by participants: 31 falls and 53 near misses (dummy falls were removed and therefore are not included in this data). Most events took place in the home environment (n = 49; 58%) followed by in public (n = 9; 10%) and outdoors (n = 8; 9%). The main cause of falls and near misses was loss of balance (n = 19; 22%) followed by trips (n = 12; 14%) and turns (n = 12; 14%). The Supplementary Table shows further details on fall characteristics.

#### Focus Groups

Two focus groups were conducted, both with 5 participants (Tab. 2 shows characteristics of participants in the focus group). All 10 participants who attended the focus groups had used the iFall app over the previous 6 months. In total, 7 men and 3 women attended a focus group meeting. The average age of focus group members was 68.3 (SD = 8.7) years, and mean disease duration was 8.10 (SD = 5.70) years; this group rated the app on the SUS at 71.8% (18.9%), classified as good.^16^

**Figure 3:**
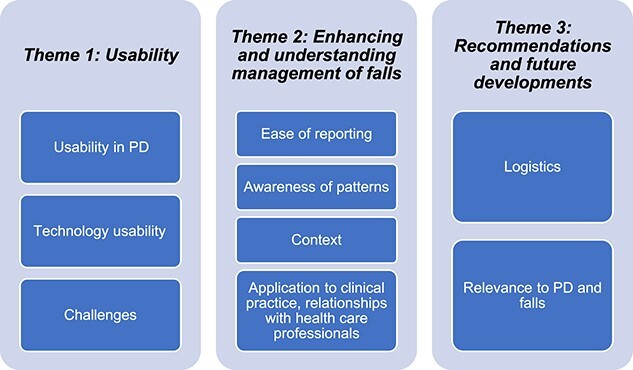
Themes and subthemes that emerged from the focus group semistructured interviews.

Three themes emerged from the interview data: usability, enhancing and understanding management of falls, and recommendations and future developments. Each of the themes contained multiple subthemes (Fig. 3). Quotes to support themes are shown in Table 3.

**Table 3:** Quotes to Support Themes and Subthemes From Focus Groups

**Theme**	**Quote**
1: Usability	
Subtheme 1A: usability in Parkinson disease	My writing is going, definitely. And certainly, the fact that you can record it, either on a phone or on a keyboard, makes it much easier. I would agree with that. (R10) What did it for me was that I can barely write now. So, keeping a note in a paper-based diary was … It’s difficult. But I can use a keyboard. So, that made it much easier for me. I don’t know about anyone else, but I can … My writing is just about illegible. Even to me. (R9) Yeah, well, my wife tends to record my falls. But if she’s not there with me, she can’t, so … And we tend to have arguments about whether I’ve fallen or not, as well. (R10) Some people want to share these things. Or you have to share with your partner or whatever … Some things … If you’ve got the whole place to yourself. So, there is that opportunity for somebody else to comment on what’s happened. (R3) It might be useful for your partner or your carer to make some comments, perhaps, about what you … If they had to help you get up or … Because there was some options—how did you get up or something—wasn’t there? If you needed another person or … And so on. And I think it would be useful for, maybe, your carer or your partner to make some comment about how you were, maybe, later on the same day. Whether your fall had a long-term impact on you or not. (R9)
Subtheme 1B: Technology usability	Yeah, for myself, it was very … Yeah, it was very straightforward. (R6) But it’s doing … It’s doing what it says on the tin. It’s doing date, time, place, reason, and so on. And that seems to me to be very, very clear. (R2) Well, I think in the case of reporting falls, yes, I would … For many years, I did the hard copy paper one. And I’ve forgotten who it was used to send me, sort of, three months at a time. And I stuck them in an envelope. But I never took a copy, so I was never able to look back. So, I think having it in this format would be very much more helpful. (R2) I used to keep my falls in my husband … My husband’s diaries. His company sends him a diary every year, and he never uses it. So, I recorded my falls in the diary. But it was easier to print it out when I went to see [Name] with it in the app. (R7) The fall happens or … You know, a near miss happens. And you get on with your life, and then you forget that you’ve fallen or you’ve had a near miss, and it doesn’t get recorded. So, from the point of view of the accuracy of the information that’s going back to clinicians, and people like yourselves … I think it improves the accuracy of it. (R8)
Subtheme 1C: Challenges with iFall usability	As I see it, there are, sort of, two main types of fall—there’s a trip … When you trip, and there’s where you lose your balance. In my case, although I’ve never had a fall … I haven’t had a near miss, really, but my balance is particularly bad recently. And I think dividing them up into one of those two options would be desirable. (R3) But there’s a limited amount of responses that you can put in. Sort of like, i.e., falling in the garden, etc. So, if you’ve tripped on the stairs, or you’ve tripped in the kitchen, there’s not that facility to … You know, to broaden the field of … You know, what might have caused the problem. (R1) I think, [Name], when you’re thinking about the number of options, it’s going to be impossible to cover everything, isn’t it? So, we probably need to … You know, for simplicity, we probably need to identify the options that are most common, and then have another box where people could perhaps type their own thing in. (R2) I had some problems early on, getting into the app, and getting it onto the phone. (R8) I’d never used Android before. And like [Name] [00:11:40], I think I prefer an iPhone version. Because that’s what I have. But it was a very good, useful tool. (R9)
2: Enhancing and understanding management of falls	
Subtheme 2A: Ease of reporting	I found it useful from the point of view that you could record a fall straightaway, and you didn’t have to remember it or record it in a written diary or anything. (R8) I think the fact that you can just record a fall immediately is very, very useful. Instead of waiting until you get home, for instance, to record a fall that you’ve had outside. I don’t know about anybody else, but I tend to forget things like that. (R10)
Subtheme 2B: Awareness of patterns	Certainly, it’s made me more aware of places where I have fallen previously. It’s … Sensitised me to the whole issue of falls. (R2) The app has helped me to be more aware, I think, of the potential hazards. And to be more careful where I put my feet. I think anticipating you, or aware that it is an issue, it is a problem … And that people were looking out for it, as it were. If there is a pattern, or there is a common denominator, then it’s worth being able to identify that. Yeah, I value the project in as much for me it’s … It’s makes me alert to the risk of falls. And I’ve had a few falls. I think it’s helped me to improve my awareness, and not only to complete the … The thing itself, but also in terms of physiotherapy. I’ve been able to focus that … With advice, obviously—to focus that on the falls and the issues surrounding falls. In my house. And it was very useful. (R3) It certainly showed me that leaning forward was a bad move. As well, when I fell forward. If I lean too far. Quite a few of my falls were caused by leaning forward for something. (R7) And it was very hard to find patterns. So, in the end, I gave up because it was doing my head in more trying to find a pattern, than actually just record. And I was recording … But if a pattern suddenly appears—great, I’ll spot it. But I never did, really. (R6) I tend to discuss with my wife, obviously, depending on what occurs. But I don’t think the app helped in that way at all. (R8)
Subtheme 2C: Context of falls	I fell backwards a lot, but it was useful to record what I was doing before. Because then I could say, well … If I was running … If I was running? Or trying to run. And I fell forwards, then it tells me not to run, doesn’t it? Yeah, that was useful. That was good. (R7) I think also we need to take account of our own domestic circumstances. I’m older, as it were, with four youngish children. The age range now from 12 through to 17. That does create hazards. The more people you’ve got around you, the more hazards there are, I think. People leaving things there. Not putting things back in the same place where you’re accustomed to finding them. And things like this … But I don’t know whether the research was taking account of the general pattern of living for each participant. (R4) I think there’s a little bit of categorisation and perhaps, as we said, a little bit of balance to be achieved with the number of options and things. (R2) The difficulty with the character spacing—there wasn’t enough sometimes to describe what happened. (R6) And then recording the falls, you know … I would like to write more. (R5)
Subtheme 2D: Application to clinical practice and relationship with health care professionals	I think that would be a very useful thing for me to take to my consultant or GP. (R1) I was at the physiotherapist yesterday. And I was showing her the app on the phone, so she was interested in it. (R5) One positive that came out of it for me, was when I went to see the Parkinson’s nurses. She looked through it and she said, “Oh, you’ve had quite a few falls.” And we talked about the … My medication routine. And she altered it slightly. At the moment, it hasn’t made a big difference very much. But it might, in time. And I saw her just before Christmas, so it’s early days yet. But she’s changed the treatment of my doses and medicine. It was a range of different things, but the falls information was quite crucial. But I would’ve had to see the diary and look for the dates and try and remember what the fall was about. But now I’ve got the information, and [Name] could take away the print … The printout. So, that was a very positive thing for me. (R7) And I don’t think it would help clinicians very much, from that point of view, to, sort of, try and collate or try and decide what caused the fall. (R8) There’s the consequences of sharing with somebody else. I have quite a different viewpoint. So, I might be trying to, sort of, emphasise a particular aspect of a fall or a trip, but actually what’s been heard is something different. I’m very sensitive at the moment, because I’m hoping to get my driving license renewed. I want to be honest. Absolutely honest. I don’t necessarily want to put myself in relation to that significantly worse for my remaining life experience. (R2)
3: Recommendations and future developments	
Subtheme 3A: Logistics of iFall	A lot of the phones or versions being used now have location to start with. And a lot of them also have accelerometers built in, or can be switched on, on the phones. Now, obviously, that could automatically pick up falls. But then that depends on how … How much money you would have to invest in … And also how big a take up. Because, obviously, it’s quite a big expense to do things like that. (R6) And I was on about the fact that I love technology which can be simple and useful—and a lot of modern phones, whether iPhones or Androids have location and the ability to switch on accelerometers or download accelerometers. So, anything that could be tweaked to make it being able to record things. But anything that could be automated to make it easier for people, I think that’s a great idea. (R8) Would there be any way of having it so that you could speak into it, and record it that way? (R9) It’s doing what it says on the tin. It’s doing date, time, place, reason, and so on. And that seems to me to be very, very clear. And as we’ve said earlier, it’s not hard to fill in the … Fill it in, once it’s up and running. You can actually make a record in a very short period of time. (R2)
Subtheme 3B: Relevance to Parkinson disease and falls	I think you should be more specific. You know what you’re saying about too many—too much information—but I think if it happens in the bathroom or the kitchen or at what time … I like to be more specific. (R10) And interpret it, and make decisions as to what medication, or what changes to medication might be useful to … To reduce the number of falls. (R8)

### Theme 1: Usability

This app is the first of its kind to provide a digital tool to report falls in people with PD. The participants discussed the usability of the app generally, but also specifically in relation to their PD-related impairments**.** Participants related their previous experiences of using technology as well as addressing the challenges to usability for this prototype of the iFall app.

#### Usability in PD

The people with PD discussed several benefits of the iFall app specific to living with PD. Although paper diaries are commonly used in PD to record falls, difficulties surrounding these were highlighted. The participants commented on challenges with writing and the impact this has on recording falls and details around their falls. The iFall app provided a solution to recording this information (Tab. 3, subtheme 1A). The roles of spouse and family were discussed in the context of the app. The participants with PD discussed their impairments and challenges with family and friends and found this app very helpful to support of day-to-day life and management. Some participants’ family members input information on their behalf. A suggestion specific for app users who value this social support was an option for family members to input falls information to provide a different perspective given their key role in the individual’s life (Tab. 3, subtheme 1A).

#### Technology Usability

The people with PD discussed the app having good basic functionality and being swift to input falls as they occurred. The simplicity and intuitive nature of the app was highlighted with frequent positive discussion of this ease of use (Tab. 3, subtheme 1B). This was supported by the results in the SUS questionnaire; participants responded that the app was easy to use (average = 4.00; min = 2, max = 5) and that they felt confident using the app (average = 4.38, min = 2, max = 5) (Fig. 2). Overall, technology was viewed positively. If people were returning their paper copies to research or health care professionals, it meant they had no record, whereas the iFall app allowed people with PD to look back and keep this record of falls with ease. Due to the simplicity and ease of use, people with PD could record a fall when this happened, and the technology improved the accuracy of falls recording in comparison to diaries.

#### Challenges With iFall Usability

A prominent area of discussion in both focus groups was the interpretation of the term “near miss”. The people with PD discussed the different meanings of this to them and this presented a challenge of when to use this categorisation. Other terms were also suggested in place of this for clarity (Tab. 3, subtheme 1C). Some people with PD found it difficult to record the circumstances surrounding the fall as it was felt options were limited and there should be more to choose from. However, the contrast to this was having too many options, which would not be desirable to people with PD. The use of a free text box was suggested to provide a useful compromise. The discussion around options within the app is supported by the results from the SUS, where some participants felt that there was some inconsistency in the app (average = 2.23, min = 1, max = 5) (Figure 2 shows further details).

There were some technological challenges with iFall. These included difficulties downloading the app, incorrect dates for recording falls and not being available on an iPhone platform. One individual did not understand the relevance of the app or how this could help with falls. There had been technological challenges from the start for this person, and their preference was then to record via paper due to the difficulties faced. However, for the most part, these technological challenges did not detract from the value of the iFall app for most people with PD. Once again, these findings were supported in the results from the SUS. Most participants felt that others would be able to learn to use the iFall app quickly, but the results were mixed depending on the individual (average = 4.00; min = 1, max = 5). Furthermore, some participants felt that they would require technical support to use the app (average = 2.00, min = 1, max = 4) (Fig. 2).

### Theme 2: Enhancing and Understanding Management of Falls

Participants considered how the falls app would enhance self-management and understanding of their own falls via raising awareness around patterns of falls and context of falls. Furthermore, participants discussed using the app alongside their health care professional to guide their assessments when visiting their health care professional. This finding was further supported by results of the SUS questionnaire with most participants agreeing that they would like to use the iFall app frequently (average = 4.08, minimum = 2, maximum = 5) (Fig. 2).

#### Ease of Reporting

The current gold standard of falls reporting is to use pen and paper diaries. In the focus group discussion, participants discussed the benefits of recording a fall straight after the event had occurred and a platform to record this on. Participants discussed the benefit of using the app, so they did not forget details around the fall (Tab. 3, subtheme 2A).

#### Awareness of Patterns

Through using the iFall app, some participants reported an increased awareness around patterns of behaviour for both falls and near misses. Participants became aware of their behavior surrounding falls and started to address potential hazards in their environment with the aim to reduce future falls (Tab. 3, subtheme 2B). However, others reported difficulties in trying to recognize patterns. One individual wanted to use the app to identify patterns but did not manage to do so in the 6 months they were using the app. Another individual preferred an alternative technique of discussing fall patterns with their spouse.

#### Context of Falls

Participants discussed greater awareness around the context of their falls when using the app. For example, the location of the fall, the cause of the fall, and landing direction of a fall. This was useful to some individuals in the focus group to help them reflect on circumstances and context surrounding a fall and how this feeds forwards into future management (Tab. 3, subtheme 2C). However, others felt that the app was quite restrictive with the categories and options provided within the prototype to record falls. Some members of the focus group also felt restricted by the character limit within the app to describe activities prior to a fall.

#### Application to Clinical Practice and the Relationship With Health Care Professionals

Alongside using iFall as a self-management tool for recording falls and noticing falls related patterns, participants also discussed the benefits of using the app alongside their health care professional. Several individuals in the group felt the app has the potential to enhance clinical consultations through providing detail of the number of falls and fall context. The recording of falls on iFall captured a detailed account of the individual’s falls history and circumstances all in 1 place to facilitate falls management planning with health care professionals (Tab. 3, subtheme 2D). Not all members of the focus group felt the app would be beneficial to use alongside health care professionals, it was felt the app may not help with identifying patterns and therefore treating falls. There were also additional concerns to sharing with health care professionals, 1 participant raised a concern around being able to share the data with other people, as they did not want the falls information to impact on other aspects of their PD, such as driver license renewal.

### Theme 3: Recommendations and Future Developments

The people with PD identified several ways that the current prototype of the iFall app could be improved for future use. Two subthemes emerged from the data, a focus on the logistics of a future prototype and how the app could provide relevance for PD and PD-related falls.

#### Logistics of iFall

Some participants discussed incorporating further technology such as voice recording and accelerometers to improve the use of the app. These suggestions demonstrated some knowledge and confidence with these potential additions and how they would further enhance the iFall experience. Voice recording aimed to provide a solution to discussions surrounding difficulties typing when living with PD (Tab. 3, subtheme 3A). This was not a unanimous view; some participants were happy with the current technology within the prototype and advised to not make the app too complicated or provide too many additions.

#### Relevance to PD and Falls

Participants discussed future recommendations for the app that were specific to PD, for example, including being more specific on the location of a fall and the ability to link in medication information that may guide further information around the pattern of their falls (Tab. 3, subtheme 3B).

## Discussion

The aim of this study was to develop a prototype of a falls reporting app (iFall) and explore usability and acceptability of the app, through coproduction with people affected by PD. A coproduced prototype was successfully developed alongside people with PD and was well received by users in our pilot usability trial. Feedback via semi-structured interviews suggested multiple potential uses for people with PD, health care professionals, and researchers with current technological barriers and future recommendations also identified.

Identifying suitable digital measurement tools for health care has gained traction in recent years, accelerated by the global pandemic.^19^^,^^20^ Digital measurement tools may improve accuracy and reduce time and specialized training, over and above subjective measures commonly used in current clinical practice and research.^21^ The development and availability of apps as a platform for digital measurement tools has particularly increased due to the low-cost and large number of smartphone users across the globe. Numerous apps are now in use for clinical populations. Specific to PD, a number of apps have been validated and available to download to monitor and treat motor and nonmotor impairments.^22^ The iFall app is the first of its kind to provide a digital solution to monitoring falls and near misses in people with PD. Considering the high incidence of falls in people with PD, the app has the potential to be used by many people with PD. Furthermore, the app is the first digital measurement tool to record near miss fall events which when used within clinical practice may help to prolong the time to first fall and reduce the number of falls in recurrent fallers. Digital health technologies provide a solution for self-management of chronic conditions, with self-monitoring a key component of self-management.^23^ Therefore, integration of the iFall app as a self-management tool and alongside health care professionals may lead to improved quality of life for people with PD and reduce burden on the health care system. In our study, participants discussed the use of incorporating further technology into the app. There is scope to integrate falls detection algorithms into an app such as iFall, which would prompt the user to record a fall detected by accelerometers. However, such algorithms require further work to refine and validate algorithms to ensure accuracy of falls detection.^24^^,^^25^ Therefore, iFall provides a useful self-management tool until such algorithms can be incorporated to advance the technology.

Usability of the iFall app was determined using the systems usability scale and via focus groups. Overall, our data indicated that participants found iFall acceptable and easy to use. Older people’s uptake of new technology can be affected by social, psychological, health and economic factors.^26–28^ While all the participants were smartphone users, not all had experience with Android devices, and some found the Android interface challenging. Users also reported issues with downloading the app, which highlighted an area for further development. Due to the nature of this research study, including the main outcomes and the research taking place virtually, we required that participants had previous experience of smartphone use. Digital inclusion must be considered when developing such tools to recognize barriers to implementation. Digital literacy has been identified as the biggest barrier for using digital health and is associated with confidence and previous occupation.^29^ To improve digital literacy and promote digital inclusion, information technologies should be designed with end-users in mind and adequate training supplied, amongst other factors.^30^ In our study, the coproduction design ensured that the app was designed with the end-user in mind, and to that end, feedback on the app interface and content was positive (eg, size of buttons, tactile feedback, fall causes, and fall location). However, several technological barriers remained. To improve accessibility and digital inclusion, we must ensure that future versions of the iFall app are available across multiple platforms (ie, Android, iOS), the app is compatible with all devices, including smartphone, tablets, and desktops and adequate resources and training are provided.

Coproduction is an activity, approach and ethos involving researchers, people with lived experience of health conditions and the public working together, sharing power and responsibility across the entirety of a project.^12^ Falls reporting currently relies on paper-based retrospective reporting which can lead to error and also be burdensome for people with PD.^7^ In the focus groups, participants commented on the use of the app compared to paper-based diaries. There was a consensus from participants that app-based reporting overcame problems with handwriting, and that previous use of paper-based diaries led to forgetting to post the diary back, leading to inaccurate data. The app also provided a log of their previous falls so they could observe their own data, which is critical for self-management. It is likely for clinical trials that use of an app such as iFall would improve accuracy compared to the current gold-standard. However, this study was completed in participants who were confident with smartphone use, and therefore a larger study observing those with varied experience of digital technology would need to be undertaken to further determine whether app-based technology would provide a more accurate solution compared to the current gold standard. Furthermore, future work would be required to compare the 2 methods of data collection to determine if 1 solution is superior. Within the study, both qualitative and quantitative research methods were used to enhance the researchers’ understanding of how people affected by PD experienced the app. O’Cathain et al provide guidance to researchers exploring uncertainties and optimizing interventions by engaging users in feasibility studies and qualitative research.^14^ They contend that this can play a role in enhancing researchers’ understanding of the users’ perspective and increase the impact of subsequent larger scale studies such as randomised controlled trials. Our study determined the usability of the app, which was found to be acceptable within our cohort. Furthermore, thematic analysis identified themes of usability, enhancing, and understanding management of falls, users’ recommendations, and future developments. Data collected from the focus group discussions enabled researchers to enhance their understanding of the participants’ views. When this data was triangulated with responses to the SUS questionnaire, user experience of the practicalities of using the app became clear and cross-cutting themes of app usability, app use in falls management and future developments of the app were identified.

The potential for health care professionals to use the app to inform clinical assessments emerged as a strong theme from the focus groups. Participants noted that they could see an advantage to this in their clinical appointments, and some participants used their smartphone to share their falls data with clinicians during our study. This use of the app may add to the data available to health care professionals on the occurrence of falls and the context around a falls event, with the potential to be embedded into health care systems.^21^ To date, health care professionals have not been involved in the coproduction of the app; however, to ensure their needs are integrated, future work will be conducted alongside clinicians. This should ensure the options within the app are suitable and generate valuable information required to guide clinical falls management.

The app also has potential for a novel outcome measure for falls frequency in clinical trials. Novel digital measurement tools have become a key focus within clinical trials, providing sensitive measures for drug development. iFall has the potential to provide a digital endpoint in clinical trials that is accessible for people with PD, providing a more accurate and sensitive measure of falls frequency. Furthermore, the app has the scope to be implemented in numerous other clinical conditions with high falls risk, alongside coproduction allowing for bespoke modifications for clinical groups.

### Strengths and Limitations

This study was the first of its kind to coproduce a falls reporting app and it was strengthened by the role of people with PD throughout the process. In our study, coherence was enhanced by the long-term engagement of people with PD in the design and management of the study. The long-term use of the app for a timescale of 6 months by a sample comprising people with PD also enhanced the internal validity of the study. Rigor was strengthened by the multi-disciplinary nature of the study team and the 2 independent coders with different clinical backgrounds. However, there were also several limitations to our study. First, our prototype was only available on the android platform and therefore this limited the population able to download and test the app. Although smartphones were provided for those who did not have an Android phone, several users were not familiar with this platform which may have limited engagement. Second, we did not specify that participants should be fallers, and although all participants engaged with the app, this again may have limited engagement with the prototype. To counteract this, we provided “dummy falls” to ensure all participants engaged with the app. Third, the sample for this study was recruited via an online network and required previous experience using a smartphone, and therefore findings may not be generalizable across the PD population. Future work should explore usability and acceptability of the app in people with PD who are not confident with online/smartphone use. Finally, the sample size for this study was relatively small; therefore, we cannot generalize the applicability of the app across the population with PD. Additional work needs to be completed with a larger sample size to further test applicability and acceptability.

## Conclusion

This is the first study to develop a falls reporting smartphone app alongside people affected by PD. A falls reporting app may provide a useful self-management and clinical tool to further inform rehabilitation around falls in PD. Furthermore, the app has the potential to be used as a digital outcome measure for research projects/clinical trials which may improve accuracy of reporting. This study identified that the current prototype had good usability in accordance with the systems usability scale. Further work is needed to develop the app and involve additional stakeholders.

## Supplementary Material

2022-0577r1_Supplementary_Figure_Study_Overview_corrected_pzad076

Supp_ifall_table_1_fall_characteristics_pzad076

2022-0577_r1_Supplementary_Appendix_iFall_Focus_Group_Interview_Structure_Final_pzad076

## Data Availability

Derived data supporting the findings of this study are available from the corresponding author on request.
