# Supplementary figures and images for: Coproduction and Usability of a Smartphone App for Falls Reporting in Parkinson Disease
Source: Phys Ther. 2023 Jun 27;104(2):pzad076. doi: 10.1093/ptj/pzad076 (PMC10851851; doi:10.1093/ptj/pzad076)

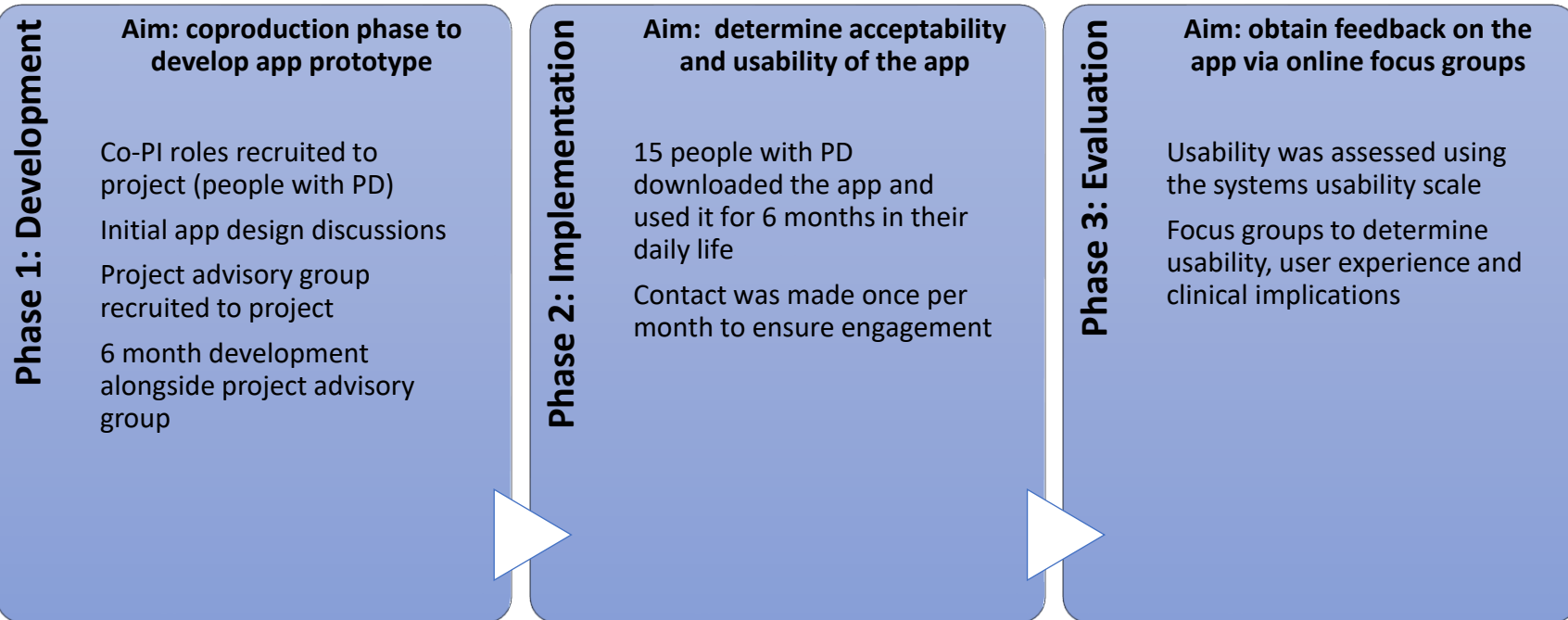

Supplement: 2022-0577r1_Supplementary_Figure_Study_Overview_corrected_pzad076 [file 2022-0577r1_supplementary_figure_study_overview_corrected_pzad076.pdf]
