# Supplementary material for: Coproduction and Usability of a Smartphone App for Falls Reporting in Parkinson Disease
Source: Phys Ther. 2023 Jun 27;104(2):pzad076. doi: 10.1093/ptj/pzad076 (PMC10851851; doi:10.1093/ptj/pzad076)
Supplement: Supp_ifall_table_1_fall_characteristics_pzad076 [file supp_ifall_table_1_fall_characteristics_pzad076.docx]

| **Event Characteristic** | **Number (percentage)** |
| --- | --- |
| **Total events logged** | 84 (100%) |
| **Number of falls logged** | 31 (37%) |
| **Number of near misses logged** | 53 (63%) |
| **Location of falls and near misses** |  |
| Home | 49 (58%) |
| Outdoors | 8 (9%) |
| Garden | 3 (3%) |
| Public | 9 (10%) |
| Hospital | 4 (4%) |
| Stairs | 5 (8%) |
| Other | 6 (8%) |
| **Cause of falls and near misses** |  |
| Loss of Balance | 19 (22%) |
| Tripping | 12 (14%) |
| Turning | 12 (14%) |
| Environment | 9 (10%) |
| Transition | 8 (9%) |
| Off Medication | 5 (6%) |
| Dizzy | 3 (3%) |
| Other | 15 (17%) |

**Supplementary Table 1**. Characteristics of fall events (falls and near-misses) during the usability trial.
