# Supplementary material for: Coproduction and Usability of a Smartphone App for Falls Reporting in Parkinson Disease
Source: Phys Ther. 2023 Jun 27;104(2):pzad076. doi: 10.1093/ptj/pzad076 (PMC10851851; doi:10.1093/ptj/pzad076)
Supplement: 2022-0577_r1_Supplementary_Appendix_iFall_Focus_Group_Interview_Structure_Final_pzad076 [file 2022-0577_r1_supplementary_appendix_ifall_focus_group_interview_structure_final_pzad076.pdf]

<u>iFall Focus Group: Interview Schedule</u>

Interview set-up

- Start audio-recording device
- Check for consent, audio and video consent

Introduction

- Introduction to research staff on project as well as project advisory group
- Thank participants for their commitment to date on the iFall project
- Overview of aims of the interview and the schedule for the focus group
- Check all participants have read the Participant Information Sheet
- Clarify that participants can withdraw at any point in the interview
- Clarify recording of the interview, and check all participants consent to this

Usability- RM/GS

- We will go round the group; please describe how you have found using the app? Has this had any impact on you? (Rosie)
- What did you like most about the app? Name one positive (Gerry)
- What did you dislike about the app? Name one negative (Gerry)
- How easy is/was using your app?
    - What (helped to make) made the information clear and understandable?
    - What (got in the way of clarity and comprehension) didn't make the information clear and understandable?
    - How did you find the font size?
    - How did you find the icon size?
- Was it easy to add your own comments in the text box?
- Was the registration process easy?
- Did the app fulfil your needs and help you to record your falls?
- Was there any medical or technical jargon used within the app that you didn't understand?
- Were you satisfied with the amount of time it takes to record a fall or near-miss on the app?

Participant experience - RM

- Did you ever forget to report a fall or near-miss on the app?
    - If yes, what were the reasons for this?
- Was the app acceptable to use following a fall or near-miss?
- Did you look at the falls reports?
    - If you looked at the reports, were the falls reports easy to understand?
- Are all the options for 'reason for your fall' on the app?
    - If not, what options were missing?
- Are all the options for injury on the app?
  If not, what is missing?

- How did you find the emotional response following the fall as an option?
- Was the app easy to use?
    - If not, how come?
- Would you recommend the use of the app to others?
- What feedback do you find useful when you are entering information? e.g., sound, tactile etc.
- Have you found any ‘bugs’ in the app or things the app can’t do?

Future Improvement- GS

- If you were designing the app, what would you do differently?
- What else do you think would be helpful to include in the app?
- Would you like to see any additional information in the reports?
    - If so, what would be useful?
- If you were to receive reminders to enter information on the app, how often would these be good to receive? (e.g., daily, weekly, monthly)
- Are there any amendments/suggestions you would make to the content/timing/visual representation of the app?

Clinical Implications- EW

- Did the app help you to better understand your falls or change your behaviour regarding your falls?
    - If yes, in what way?
    - If no, can you elaborate on why not?
- Who do you think would benefit from using the app?
- Would you use this app as part of a research project?
- Would you use this app to take discuss details of your falls and near-misses with your doctor/physiotherapist?
- Does your Doctor/Physiotherapist know you have been using the app?
    - If yes, how would you describe their reaction?
    - Do you feel encouraged by your health care professional to use the app?
